# Sentinel lymph node biopsy with carbon dye in endometrial cancer: a single center, prospective cohort study

**DOI:** 10.1007/s00464-023-10662-1

**Published:** 2024-01-30

**Authors:** Yagmur Minareci, Hamdullah Sozen, Buket Altinkara Hacioglu, Huseyin Oguz Yuvanc, Samet Topuz, Mehmet Yavuz Salihoglu

**Affiliations:** 1 Division of Gynecologic Oncology, Department of Gynecology and Obstetrics, Faculty of Medicine, Istanbul Medical Faculty, Istanbul University, Turgut Ozal Millet Cd, Monoblok Binasi, No:118, Zemin Kat, Jinekolojik Onkoloji Poliklinigi, Capa, Fatih, 34093 Istanbul, Turkey; 2 Department of Pathology, Eskisehir City Hospital, Eskisehir, Turkey; 3 Department of Gynecology and Obstetrics, Eskisehir City Hospital, Eskisehir, Turkey

**Keywords:** Carbon dye, Sentinel lymph node biopsy, Endometrial cancer, Carbon nanoparticles, Lymph node metastasis

## Abstract

**Background:**

Many agents have been used for the detection of sentinel lymph nodes in endometrial cancer. Carbon dye, which has a strong staining ability and high contrast due to its dark black color, identifies the lymph nodes efficiently. Our aim was to evaluate the safety and efficacy of carbon dye for the detection of sentinel lymph nodes in endometrial cancer.

**Methods:**

We conducted a single-center, prospective, cohort study in 89 patients with early-stage endometrial cancer between September 2021 and August 2022. The procedure was performed under laparoscopy.

**Results:**

The sensitivity and negative predictive value of the sentinel lymph node biopsy (SLNB) with carbon dye injection were 83.3% and 98.8%, respectively. Mapping identified at least one sentinel lymph node in 84 patients (94.4%) on one pelvic side and at least one sentinel lymph node in 73 patients (82.0%) on each pelvic side out of 89 patients. In addition, no carbon dye-associated allergic reaction was detected.

**Conclusion:**

Carbon dye is a non-allergenic, inexpensive, and effective agent for SLNB with a satisfying sensitivity and a negative predictive value. In addition, both unilateral and bilateral detection rates were sufficient. Accordingly, carbon dye may be a promising tracer and a considerable option, particularly for low-income countries.

**Supplementary Information:**

The online version contains supplementary material available at 10.1007/s00464-023-10662-1.

Endometrial cancer is the fourth most common cancer in women after breast, lung and colorectal cancer in developed countries. Thus, it is the most common gynecological cancer. Fortunately, most patients are diagnosed at an early-stage and except in special circumstances, conventional surgical treatment of early-stage endometrial cancer consists of hysterectomy, bilateral salpingooferectomy, and systematic lymphadenectomy [1]. Lymphadenectomy determines the disease stage and prognosis, and clarifies the decision for adjuvant therapy. On the other hand, systematic pelvic and para-aortic lymphadenectomy is associated with major complications, including life-threatening great vessel injury and lower extremity lymphedema.

Sentinel lymph node biopsy (SLNB) may be defined as a minimally invasive procedure compared to systematic lymphadenectomy. Besides, SLNB has currently been known to not compromise the progression-free and overall survival [2]. In addition, SLNB not only reduces the rate of major surgical complications, but also shortens the operation time. Furthermore, SLNB facilitates the detection of small-volume disease, which can not be detected in systematic lymphadenectomy [3].

Many agents have been used for the detection of SLNB in endometrial cancer. Blue dyes (isosulfan blue, methylene blue, patent blue) are the best-known and most commonly used agents and require no special equipment. In addition, the simultaneous use of radiolabeled colloid technetium 99 (Tc99) with blue dyes further increases the detection rate of sentinel lymph nodes. However, a gamma counter is required in the perioperative use of Tc99. Indocyanine green (ICG) emits a fluorescent signal in the near-infrared (NIR) light range and needs a NIR camera and related equipment. ICG has been shown superior to blue dye and Tc99 combination for the detection of SLNB [4, 5]. On the other hand, carbon dye, consisting of carbon nanoparticles with a diameter of 150 μm, enters the lymphatic circulation by macrophages and is mainly excreted through the gastrointestinal tract [6]. Carbon dye, which has a strong staining ability and high contrast capacity due to its dark black color, enables the lymph nodes efficiently identified. Moreover, carbon dye metabolization is prolonged and may be detected in vivo months after the injection [7]. Many researchers have reported that carbon dye has a high safety profile with no significant adverse effects [8–10]. The first report on the carbon dye was about its use as an adjunct to isosulfan blue in detecting sentinel lymph nodes in patients with colon cancer [11]. Thereafter, carbon dye has gained acceptance among general surgeons in SLNB procedures. The present study is one of the few studies using carbon dye alone as a tracer during the SLNB procedure in endometrial cancer [6, 12, 13]. Therefore, the objective of the present study was to evaluate the safety and efficacy of carbon dye for the detection of sentinel lymph nodes in endometrial cancer patients.

## Materials and methods

The present study was conducted at Eskisehir City Hospital, Clinic of Gynecologic Oncology in collaboration with Istanbul University, Istanbul Medical Faculty, Department of Gynecologic Oncology, and it was designed as a single-center, prospective cohort study. The instutional review board and ethics committee approved our study protocol (ethics number: 1671, date: 11/2020). All the written informed consents were obtained from the patients. The extent of myometrial and/or cervical invasion was evaluated using the magnetic resonance imaging (MRI). All patients underwent preoperative positron emission tomography/computed tomography scanning. The inclusion criteria were the (a) presence of documented endometrial cancer in pathology specimens obtained by endometrial sampling, regardless of histological type and grade, (b) presence of early-stage endometrial cancer (according to the International Federation of Gynecology and Obstetrics (FIGO) 2009 classification, stage I-II). The exclusion criteria were the (a) presence of any other synchronous cancer, (b) presence of clinically and/or radiologically proven advanced FIGO stage (III-IV), (c) medically inoperable patients due to performance status and/or co-morbidity, (d) patients received chemotherapy and/or radiotherapy and/or other treatment strategies such as targeted therapies prior to participation,

Carbon dye was slowly injected into the uterine cervix at 3 and 9 o’clock to 1–2 mm depth for superficial and to 1–2 cm depth for deep injection via insulin syringe after draping of the patient. A total of 4 mL carbon dye was injected per patient (1 mL per cardinal point as follows: left superficial, left deep, right superficial, right deep). The tracer injection was performed after all ports were placed and just before the uterine manipulator (Clermont Ferrand uterine manipulator, Karl Storz, Tutlingen, Germany) was inserted into the cervical canal. Accordingly, the SLNB procedure was started within 15 min. The Karl Storz (Tutlingen, Germany) full HD unit was used for endoscopic imaging in all surgical procedures. A sufficient mapping was defined by detecting a lymph channel draining from the cervix to a potential lymph node on at least one side of the pelvis. After following the black-stained lymphatic channels under the laparoscope, the first stained lymph node was accepted as the sentinel lymph node, and the next node was noted as secondary. Then, the detected sentinel lymph nodes were harvested and marked for the location (Video 1). When no sentinel lymph node(s) were detected on one side of the pelvis, lymphatic channels were carefully followed, including all the possible drainage pathways. However, reinjection of the tracer was not performed in case of failure. Additionally, if no sentinel lymph node(s) were detected on any side of the pelvis, the para-aortic area was examined systematically from caudal to cranial following the possible lymphatic pathway. Completion of the bilateral systematic lymphadenectomy (removal of all non-sentinel lymph nodes) was then performed. Regardless of sentinel lymph node detection status, bilateral pelvic lymphadenectomy was performed for all patients without exception. However, paraaortic lymphadenectomy was not performed if there were low-risk factors for para-aortic nodal involvement or technical unfeasibility. Otherwise, para-aortic lymphadenectomy was routinely performed. According to pre-operative MRI and pathological report of endometrial sampling, we defined the patients with low-risk factors for para-aortic nodal involvement as those with grade 1 or 2 disease, myometrial invasion of less than 50%, and tumor diameter of less than 2 cm [14]. In addition, frozen sectioning was not performed to exclude para-aortic lymphadenectomy for any risk factor groups of para-aortic nodal involvement. All operations were performed by the same senior surgeon (YM) who was specially trained in laparoscopy. The graphical data of the sentinel lymph node location was marked just after the surgery. The data maps included the descriptive numerical labeling of the sentinel lymph nodes according to the order in which they were located on the lymphatic channel. Locations of lymph nodes were grouped as the obturator, external iliac, common iliac, presacral, para-aortic and other nodes. All the harvested sentinel lymph nodes confirmed the presence of nodal tissue via hematoxylin–eosin staining. The sentinel lymph nodes were evaluated using the ultrastaging according to the standard protocol. Sentinel lymph nodes were sliced at 3 mm intervals. However, if the nodes were ≤ 0.5 cm in diameter, they were bisected. Two paraffin embedded slides were made from each section, 50 µm apart. The first slide was stained for hematoxylin and eosin (H&E) and the second was reserved for immunohistochemistry staining. In case of the identification of no metastatic disease in the first H&E slide, the second slide was stained for pan-cytokeratin AE1 and AE3. Metastatic nodal disease was categorized into three groups. Macrometastases were defined as foci of metastasis ≥ 2 mm, micrometastases were defined as foci of metastasis between 2 and 0.2 mm, isolated tumor cells were defined as foci of metastasis < 2 mm or individual cells positive stained for pancytokeratin. An experienced pathologist (BAH) examined all the cases. The primary endpoint was to determine the diagnostic accuracy and safety of carbon dye in detecting the sentinel lymph node(s) during lymphatic mapping. The secondary endpoint was to determine the sensitivity and negative predictive value of sentinel lymph node specimens in the detection of metastatic disease during lymphatic mapping.

Sensitivity was defined as the proportion of women with positive nodal disease who underwent successful lymphatic mapping (either unilateral or bilateral) and had metastatic disease correctly detected in the specimen of the sentinel lymph node. The negative predictive value was defined as the proportion of negative sentinel lymph node specimens that were related to specimens of negative non-sentinel lymph nodes. Based on the hypothesis that if a sentinel lymph node is positive, diagnosis of lymph node metastasis is clear, the specificity and positive predictive value were not reported in the present study. The bilateral detection rate was calculated as the number of patients with bilaterally detected sentinel lymph nodes divided by the total number of patients who underwent SLNB and the unilateral detection rate was calculated as the number of patients with unilaterally detected sentinel lymph nodes divided by the total number of patients who underwent SLNB.

## Results

**Fig. 1:**
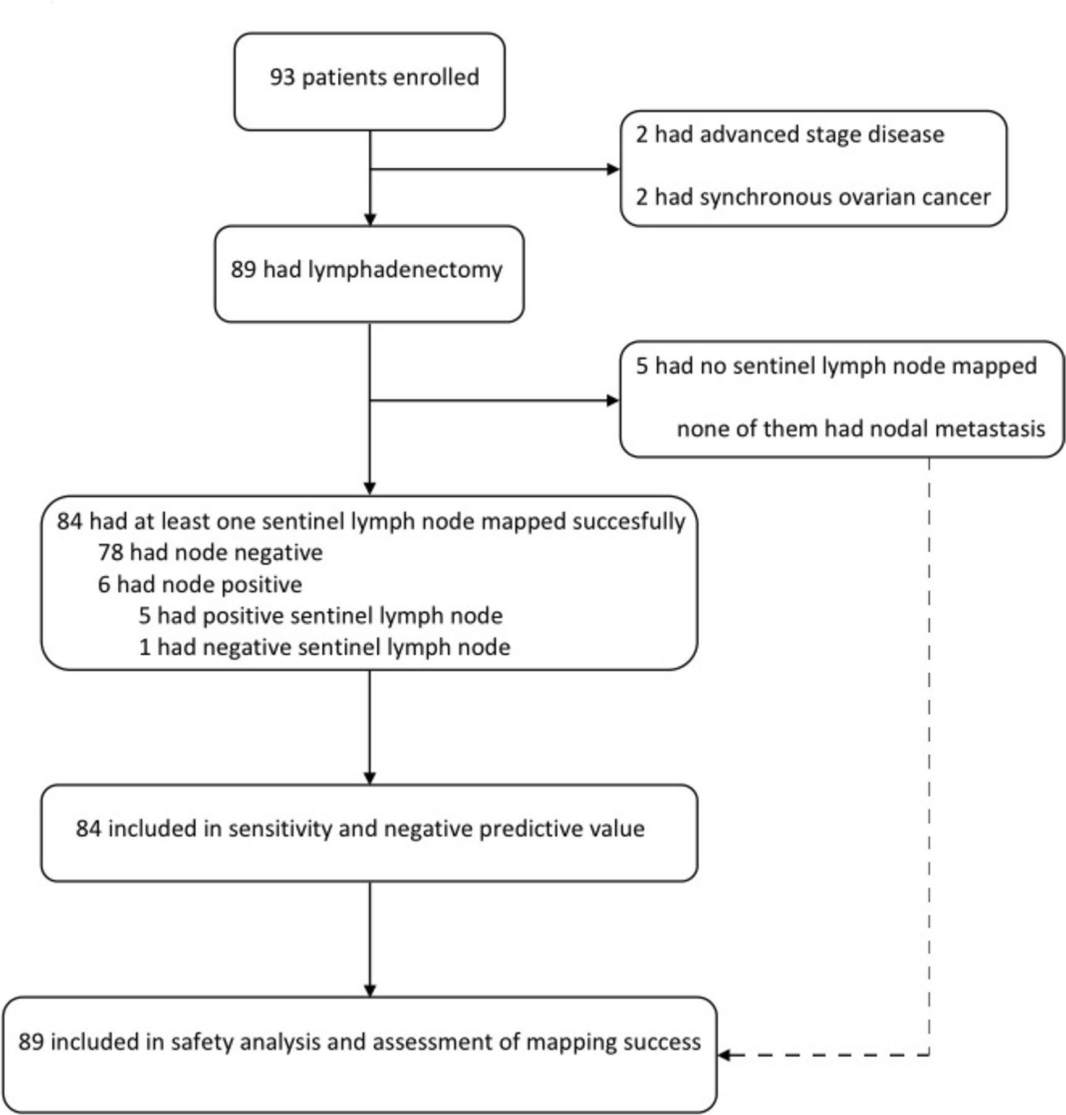
Flow diagram of the study

A total of 93 patients with newly diagnosed endometrial cancer and related clinicopathological data were prospectively collected for the present study between September 2021 and August 2022. Of the 93 patients, four were excluded due to extensive disease or concomitant gynecological cancer based on the final pathology report, and 89 patients met our inclusion criteria. Figure 1 shows the interventions for the patients. Of the 89 patients, 61 were stage IA, 20 were stage IB, five were IIIC1, two were II, and one was IIIC2. The median age at diagnosis was 58 (interquartile range 51–64) years.The patient characteristics are shown in Table 1. Perioperatively, none of the surgeries converted into laparotomy. However, due to the large uterine dimensions, uterus was extracted via a Pfannenstiel incision in two cases.

**Table 1:** Patient characteristics

Postoperative stage	*n* (%)
IA	61 (68.5%)
IB	20 (22.5%)
II	2 (2.2%)
IIIA	0 (0%)
IIIB	0 (0%)
IIIC1	5 (5.7%)
IIIC2	1 (1.1%)
IV	0 (0%)

Postoperative histology	*n* (%)
Endometrioid	77 (86.5%)
Grade 1	26 (33.8%)
Grade 2	39 (50.7%)
Grade 3	12 (15.5%)
Serous	4 (4.5%)
Carcinosarcoma	6 (6.7%)
Clear cell	0 (0%)
Undifferentiated	2 (2.3%)

Myometrial invasion	*n* (%)
None	20 (22.5%)
< 50%	45 (50.5%)
≥ 50%	24 (27.0%)

LVSI	*n* (%)
Negative	79 (88.8%)
Positive	10 (11.2%)

Lower uterine segment involvement	*n* (%)
Negative	60 (67.4%)
Positive	29 (32.6%)

Tumor size (centimeters)	*n* (%)
< 2	18 (20.2 %)
≥ 2 and < 4	42 (47.2%)
≥ 4	29 (32.6%)

Age (years)	*n* (%)
< 40	1 (1.1%)
≥ 40 and < 60	53 (59.6%)
≥ 60	35 (39.3%)

BMI (kg/m^2^)	*n* (%)
< 18.5	0 (0%)
≥ 18.5 and < 30	27 (30.3%)
≥ 30	62 (69.7%)

*LVSI* lymphovascular space invasion, *BMI* body mass index

**Fig. 2:**
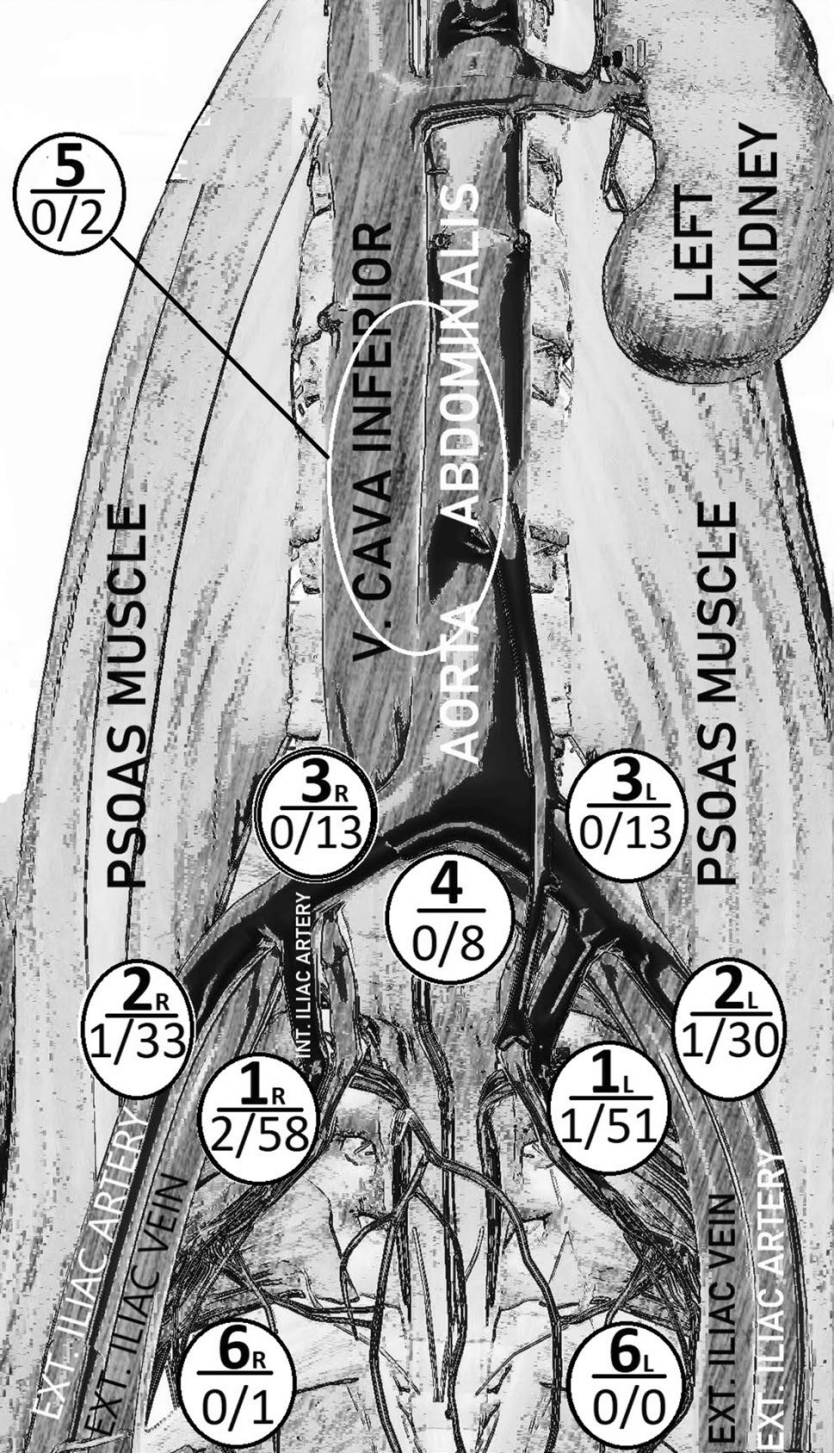
Anatomic locations of sentinel lymph nodes. Numbers: Positive sentinel lymph nodes**/**successfully mapped sentinel lymph nodes per location. 1_R_: Right obturator fossa (27.7%), 1_L_: Left obturator fossa (24.4%), 2_R_: Right external iliac area (15.8%), 2_L_: Left external iliac area (14.4%), 3_R_: Right common iliac area (6.2%), 3_L_: Left common iliac area (6.2%), 4: Presacral area (3.8%), 5: Para-aortic area (1%), 6_R_: Right parametrial area (0.5%), 6_L_: Left parametrial area (0%), Ext: External, Int: Internal

Of the 89 patients, bilateral pelvic lymphadenectomy and para-aortic lymphadenectomy were performed in 89 (100%) and 36 (40.5%), respectively. In addition, of the 23 patients with high-grade tumors, 14 (60.7%) had para-aortic lymphadenectomy. Mapping identified at least one sentinel lymph node in 84 patients (94.4%) on one pelvic side and at least one sentinel lymph node in 73 patients (82.0%) on both pelvic side (Table 2). Among the 89 patients, five had positive sentinel nodes. Of the five patients, three had micrometastasis, and the remaining two had macrometastases in one and isolated tumor cells in the other. Moreover, five patients (100%) had the disease correctly identified in the SLNB procedure. Two hundred nine sentinel lymph nodes were harvested from eight different locations. Three of the five metastasis-positive sentinel lymph nodes were detected in the obturator fossa and two in the external iliac areas (Fig. 2).

**Table 2:** Surgical results in patients who underwent staging lymphadenectomy

	Number of patients (*n* = 89)
Pelvic lymphadenectomy	89 (100%)
Pelvic + para-aortic lymphadenectomy	36 (40.5%)
Successful unilateral mapping of sentinel lymph nodes	84 (94.4%)
Successful bilateral mapping of sentinel lymph nodes	73 (82.0%)
Median number of sentinel lymph nodes harvested	1 (1–1)
Median number of pelvic lymph nodes harvested	17 (12–22)
Median number of para-aortic lymph nodes harvested	7 (2–9)
Total number of sentinel lymph nodes harvested	209
Total number of pelvic lymph nodes harvested	1508
Total number of para-aortic lymph nodes harvested	204
Number of patients with > 10 lymph nodes harvested during bilateral pelvic lymphadenectomy	83 (93.3%)

Data are *n* (%), median (interquartile range 25th–75th)

The sensitivity of the SLNB with carbon dye in detecting total nodal metastatic disease was 83.3%. However, if the sensitivity is calculated by considering pelvic lymph nodes only, excluding the para-aortic ones, the sensitivity increases to 100%. On the other hand, of the 84 patients with negative sentinel lymph nodes, all 84 had truly negative non-sentinel lymph nodes in pelvic lymphadenectomy specimens. However, one patient with negative SLNB had an isolated positive para-aortic lymph node with micrometastasis resulting in a negative predictive value of 98.8% (Table 3). In this particular case, there were no enlarged lymph nodes in the pelvic or para-aortic region at the time of surgery. Interestingly, the pathology report revealed that the 59-year-old patient had a 35-mm tumor in diameter with grade 3 endometrioid histology and 25% myometrial invasion with substantial LVSI. Patients with risk factors for lymphatic metastasis were detailed in Table 4. All patients with positive nodal disease had at least one known risk factor for lymph node metastasis. The presence of a tumor diameter ≥ 2 cm was the common risk factor in all sentinel lymph node-positive patients.

**Table 3:** Sensitivity and negative predictive value data of SLNB with carbon dye (*n* = 89)

	True positive Nodes	True negative Nodes
Positive sentinel lymph nodes	5	0
Negative sentinel lymph nodes	1^**a**^	83

^a^One patient had isolated para-aortic lymph node metastasis

*SLNB* sentinel lymph node biopsy

**Table 4:** Risk factors for lymphatic metastasis

Risk factor	Node negative (*n* = 83)	Node positive (*n* = 6)
Tumor size		
< 2 cm	22 (26.5%)	0 (0%)
≥ 2 cm	61 (73.5%)	6 (100%)
Grade		
1 or 2	63 (75.9%)	3 (50.0%)
3^a^	20 (24.1%)	3 (50.0%)
Lymphovascular space invasion		
Negative	76 (91.6%)	3 (50.0%)
Positive	7 (8.4%)	3 (50.0%)
Myometrial invasion		
< 50%	66 (79.5%)	3 (50.0%)
≥ 50%	17 (20.5%)	3 (50.0%)
Lower uterine segment involvement		
Negative	57 (68.7%)	3 (50.0%)
Positive	26 (31.3%)	3 (50.0%)
Age (years)		
< 50	12 (14.5%)	1 (16.7%)
51–69	61 (73.5%)	2 (33.3%)
≥ 70	10 (12.0%)	3 (50.0%)

^a^Grade 3 tumors include serous, undifferentiated, carcinosarcoma histology, and endometiroid histology with grade 3

Finally, two minor complications unrelated to carbon dye injection were detected during surgery. In addition, there was a major complication, including a vena cava inferior injury during para-aortic lymphadenectomy. Fortunately, the problem was solved rapidly via laparoscopic suturing of the vein with 5/0 prolene, and no other intervention was required perioperatively, including blood transfusion. Moreover, according to the Clavien Dindo Classification, no postoperative grade 3 or 4 complications were recorded. On the other hand, no carbon dye injection-related complication was reported. The only remarkable finding was that the vaginal cuff was still stained black (tattoo) at the 3rd and 6th months of postoperative follow-up. However, no complaints were reported due to the ‘tattoo’ of the vaginal cuff during the follow-up periods.

## Discussion

The results of the present study showed that carbon dye was an easy-to-use, inexpensive, and effective agent for SLNB with a satisfying sensitivity and a negative predictive value. Moreover, we achieved high unilateral and bilateral detection rates with carbon dye during SLNB. Accordingly, carbon dye may be a promising tracer with no need for expensive equipment and time-consuming nuclear imaging procedures, particularly for low-income countries. Finally, we observed no adverse events, including allergic reactions, with the injection of carbon dye, and we consider that black staining (tattoo) of the vaginal cuff was not disturbing for patients. On the other hand, our study powered that sentinel lymph node biopsy is equivalent to lymphadenectomy in the staging of endometrial cancer. The results of the current study are also consistent with the prospective and retrospective series in endometrial cancer [15–18].

Since the uterus is a midline organ, achieving a highly bilateral detection rate is critical to avoid the dissection of the pelvic lymph nodes unilaterally. Accordingly, we believe that a high bilateral detection rate causes a reduced number of unilateral and bilateral systematic pelvic lymphadenectomies, potentially reducing peri- and postoperative morbidity. In the prospectively designed FIRES study, which included 340 patients undergoing pelvic lymphadenectomy due to endometrial cancer, Rossi et al. reported that successful unilateral and bilateral mapping of sentinel lymph nodes were reported in 293 (86%) and 177 (52%) patients, respectively. In a recent review in which the Clinical Practice Committee of the Society of Gynecologic Oncology reviewed the current literature, Holloway et al. concluded that cervical injection of ICG has similar rates of successful mapping results to those of radiocolloid Tc99 and blue dye combination (for overall detection rate 95% vs %86, and bilateral detection rate 66% vs 57% respectively) [1]. On the other hand, in the prospective study of Zao et al., which included 115 patients with endometrial cancer, unilateral and bilateral detection rates with carbon dye were 96.5% and 83.5%, respectively [13]. Recently, Tao et al. conducted a study using carbon dye for SLNB in 54 patients with early-stage endometrial cancer, and the authors reported unilateral and bilateral detection rates as 70.4% and 40.7%, respectively [12]. In the present study, we calculated the unilateral and bilateral sentinel lymph node detection rates with carbon dye as 94.4% and 82%, respectively. Finally, our study revealed that carbon dye is an inexpensive and readily available tracer with high visibility to the naked eye. As in Turkey, many hospitals in low-income countries do not have expensive NIR cameras and related equipment. In addition, a limited number of hospitals have nuclear medicine units for the detection of radiolabeled colloid Tc99. In these circumstances, we consider that the administration of carbon dye during SLNB in patients with endometrial cancer may be a considerable option.

In the FIRES study, Rossi et al. reported that the sensitivity of SLNB with ICG was 97.2%. However, among the 258 patients with negative sentinel lymph nodes, one patient had a positive non-sentinel pelvic lymph node, resulting in a negative predictive value of 99.6 [16]. In the retrospective analysis including 125 endometrial cancer patients, Ballester et al. used Tc99 and blue dye combination as dual tracers and gave the results of sensitivity and NPV as 84% and 97%, respectively [17]. In the present study, we achieved an acceptable sensitivity and a high negative predictive value of 83.3% and 98.8% in detecting total metastatic nodal disease, respectively. However, we calculated the sensitivity by considering all harvested nodes. On the other hand, if we calculated by excluding paraartic nodes and considering only pelvic nodes, the sensitivity reached 100%. Indeed, the detection of para-aortic sentinel lymph nodes after cervical injection is significantly lower compared to other sites of injection [19, 20]. The possible explanation for this situation may be that the para-aortic nodal metastases occur through a different pathway. To date, three different uterine lymphatic drainage pathways to nodal structures have been identified, including the lower, upper paracervical, and infundibulopelvic pathways. While the lower and upper paracervical pathways are mainly responsible for pelvic lymph node metastases, infundibulopelvic pathway (IPP) is considered to responsible for isolated para-aortic metastases [13]. Accordingly, tumor cells may have metastasized to the para-aortic area, bypassing the pelvic lymph nodes using the IPP route. Thus, the presence of negative pelvic sentinel lymph nodes and positive isolated para-aortic lymph node after cervical injection of the carbon dye may be explained by this hypothesis. In the same vein, there are some novel reports in the literature on the active search for the para-aortic sentinel lymph nodes through the IPP. In light of various meta-analyses, we injected carbon dye into the cervix due to its easy applicability and high detection rates of pelvic sentinel lymph nodes [21–26].

We administered a total of 4 ml of carbon dye, with 1 ml of each injection point in the present study. Accordingly, we observed that carbon dye injected at this dose caused high levels of staining in the pelvic and para-aortic lymph nodes within 10 and 20 min, respectively. The most significant advantage of carbon dye is that it can be detected immediately due to its rapid penetration into the tissue and unique lymphatic system affinity. These findings are in line with previously published studies [7, 27]. In addition, carbon dye is easily recognized by the naked eye. Therefore, no specialized equipment is needed. Finally, we found no adverse events such as swelling, rash, or other allergic reactions during and after the injection, and the only remarkable postoperative finding was the isolated staining of the vaginal cuff. However, in contrast to the cosmetic dissatisfaction observed due to carbon dye injection for SLNB during breast surgery, our cohort had no complaints of ‘tattoos’ on the vaginal cuff [28–30].

Our study has some limitations. First, the present study evaluated the carbon dye only during the SLNB procedure. Unfortunately, we did not compare carbon dye head to head with ICG, Tc99 + blue dye, and other tracers. Second, a limited number of patients were included in the study cohort. A larger sample size would have yielded more reliable results. Third, the same senior surgeon performed the SLNB procedure in all operations. The results would undoubtedly have a wider variability if multiple surgeons were involved. Lastly, we did not assess the applicability of carbon dye for aortic sentinel lymph node detection, which was another disadvantage.

In conclusion, carbon dye is a non-allergenic, inexpensive, and effective agent for SLNB with a satisfying sensitivity and a negative predictive value. In addition, unilateral and bilateral detection rates were sufficient. Accordingly, carbon dye may be a promising tracer and a considerable option, particularly for low-income countries. Further work should be done to evaluate the efficacy and safety of the carbon dye in endometrial cancer.

### Supplementary Information

Below is the link to the electronic supplementary material.

Supplementary file1 **Video 1**: Sentinel lymph node mapping using carbon dye in endometrial cancer: After the cervical injection of carbon dye, the surgeon starts to dissect the right pelvic area to find the sentinel lymph node(s). During the video, the right common iliac artery and right ureter are seen together. Finally, a sentinel lymph node is identified very clearly between the ureter and artery. (MP4 143826 KB)
